# Study on the mechanism of family resilience on loneliness in older adults with stroke

**DOI:** 10.3389/fpsyg.2025.1558363

**Published:** 2025-05-29

**Authors:** Hui Ju, Yanli Dong, Ju Liu, Jing Mu, Lei Ge, Jie Chen

**Affiliations:** ^1^Department of Emergency, People's Hospital of Rizhao, Rizhao, Shandong, China; ^2^School of Nursing, Sun Yat-sen University, Guangzhou, Guangdong, China; ^3^Department of Nursing, People's Hospital of Rizhao, Rizhao, Shandong, China; ^4^Department of Radiation Oncology, Sun Yat-sen Memorial Hospital, Sun Yat-sen University, Guangzhou, Guangdong, China

**Keywords:** older adults with stroke, loneliness, family resilience, social support, family function, psychological capital

## Abstract

**Objective:**

This study examines loneliness in stroke patients who were ≥60 years of age, identifying key factors such as family resilience, social support, family function, and psychological capital.

**Methods:**

We sampled 234 stroke patients who were ≥60 years of age. These patients were diagnosed with stroke (ischemic stroke, hemorrhagic stroke) by the hospital and their condition is stable in the recovery period. Data were collected using the UCLA Loneliness Scale, Family Resilience Assessment scale, Positive Psychological Capital scale, Family APGAR Index, and Social Support Rating Scale. Using one-way analysis of variance and multiple linear regression equation to analyze the influencing factors of loneliness in older adults with stroke; Construct a structural equation model to explore the pathways of social support, family function and psychological capital in the relationship between family resilience and loneliness.

**Results:**

The average loneliness score among older adults with stroke was (35.07 ± 15.24). Factors affecting loneliness included income, activity level, community resources, social participation, and illness duration. Pearson analysis showed significant negative correlations between loneliness and family resilience (*r* = −0.738, *P* < 0.01), social support (*r* = −0.715, *P* < 0.01), family function (*r* = −0.745, *P* < 0.01), and psychological capital (*r* = −0.684, *P* < 0.01). Family resilience has a direct negative predictive effect on loneliness (β = −0.342, *P* < 0.001). Social support, family functioning, and psychological capital play a chain mediating effect between family resilience and loneliness, with a mediating effect value of −0.436, accounting for 56.04% of the total effect.

**Conclusion:**

Loneliness in elderly stroke patients is moderate. Strengthening family resilience and support systems can effectively reduce loneliness.

## 1 Introduction

Stroke is an acute neurological disorder resulting from either the blockage or rupture of cerebral blood vessels. As population aging intensifies, stroke has emerged as a leading factor contributing to the global disease burden (Li X. Y. et al., 2024). Research suggests that the likelihood of experiencing a stroke rises with age, particularly among individuals over 60, who are at increased risk, while those over 70 are even more susceptible to severe disability or death. This places substantial economic and caregiving pressures on both families and society (Collaborators GBDS, 2021).

Studies have shown that stroke patients are highly prone to suffering from varying degrees of physical, language, and cognitive impairments (VanGilder et al., 2020; Elendu et al., 2023; Nakawah and Lai, 2016). These issues can lead to feelings of stigma and self blame among patients, who then actively or passively reduce their interactions with family and society, thereby experiencing higher levels of loneliness (Guo et al., 2024; Zhu et al., 2019; Fan et al., 2023; Huang et al., 2024; Hughes and Cummings, 2020). Loneliness, defined as a subjective emotional distress caused by the gap between expected and actual social connections (Seemann, 2022), can hasten physical decline in the elderly and is linked to moderate to severe depression, cognitive decline, and poor self-perceived health (Odaci Comertoglu et al., 2024). In severe cases, loneliness can directly affect the nervous system, increasing the risk of stroke recurrence by 40% (Cené et al., 2022), which significantly worsens their rehabilitation outcomes.

According to the theory of social ecosystem, psychological capital, family functioning, and social support are the influencing factors of loneliness in stroke patients (Yang et al., 2025). Psychological capital, family function, and social support are closely related to the three elements of family belief system, family communication process, and family organizational model in Walsh's family resilience framework model (Walsh, 2016). It can be inferred that there is a certain correlation between family resilience and loneliness, which can indirectly affect the loneliness of stroke patients through the effects of psychological capital, family function, and social support.

**Figure 1:**
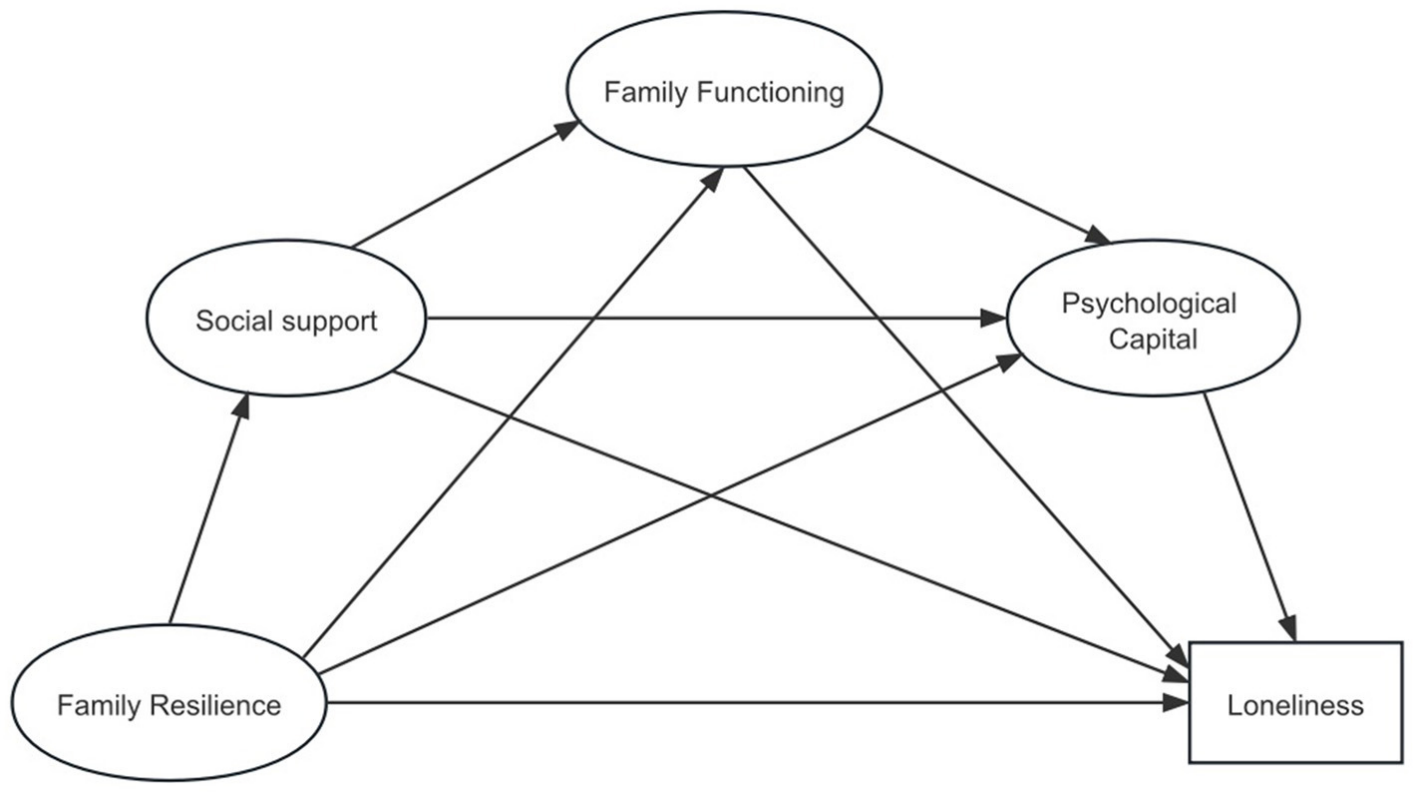
Hypothetical model.

Family resilience refers to a family's ability to adapt positively when confronted with adversity, characterized by constructive behavior patterns that are strongly linked to individual psychological resources, family functioning, and the availability of social support (Walsh, 2003; Li et al., 2019; Wong et al., 2019; Zhang et al., 2022). Strong psychological capital, healthy family dynamics, and high levels of social support are crucial for reducing patients' psychological distress and promoting their reintegration into society. Research has found that strong psychological capital can help patients maintain a relatively stable mentality when facing pressure and difficulties caused by illness (Parviniannasab et al., 2022). Family functioning can help patients enhance their psychological resilience and subjective wellbeing, and high-level social support can significantly improve their mental health (Coty and Wallston, 2010; Zhou and Kulick, 2023). These can effectively enhance their positive coping strategies for illness, and reduce loneliness. It is therefore suggested that family resilience could play a vital role in alleviating loneliness among older adults with stroke. However, the specific mechanisms through which it achieves this outcome is still mostly unclear. According to the theory of social ecosystem and the theory of family elasticity, combined with the literature review, we proposed the following assumptions and developed the model (Figure 1).

This study mainly discusses the current situation and influencing factors of loneliness in older adults with stroke, and constructs a Structural Equation Model (SEM) using psychological capital, family function, and social support as a mediating factor to explore the relationship between family resilience and loneliness. Provide new research insights and theoretical guidance for alleviating loneliness in older adults with stroke from the perspective of improving family resilience.

## 2 Object and method

### 2.1 Research subjects

We used convenience sampling to select 234 older adults with stroke discharged from the Rizhao Stroke Diagnosis and Treatment Center between December 2023 and June 2024. The study was approved by the Medical Ethics Committee of Rizhao People's Hospital (Ethical Approval Number: 2024-MR-40-01). The study was performed in accordance with the STROBE guidelines. Trial registration: Chinese Clinical Trial Registry Identifier: MR 37-24-043662.

Included were: (1) Patients diagnosed with stroke (both hemorrhagic and ischemic stroke) confirmed by cranial CT or MRI, in accordance with the diagnostic criteria of the Chinese Clinical Management Guidelines for Cerebrovascular Disease (Lou et al., 2020). (2) Patients with stroke aged 60 years or older. (3) Patients with stable stroke conditions and in the recovery phase. Excluded were: (1) Patients with mental illness, severe cognitive or memory impairment. (2) Patients with a history of significant traumatic events (such as bereavement, severe accidents, or cancer diagnosis) in the past 6 months.

### 2.2 Sample size

This cross-sectional survey investigates the prevalence of loneliness among older adults with stroke in a specific region, employing a two-tailed test with a significance level of α = 0.05. Based on multiple studies, ~40–66% of older adults with stroke experience feelings of loneliness (Liu X. et al., 2021; Byrne et al., 2022; Hollands et al., 2024; Zhang M. Y. et al., 2023). Structural equation modeling (SEM) requires large samples for computation because large samples can provide more stable parameter estimates, enhance the power of statistical tests, and better meet the assumptions of data distribution, thereby ensuring the reliability and accuracy of the model. Consequently, the anticipated prevalence *(P*) was set at 40%, with an allowable error (δ) of 1/6 of *P*. Utilizing the sample size calculation formula for proportions (Verma et al., 2024), the required sample size was determined to be 210 participants. To account for an estimated 10% rate of invalid questionnaires, the study aimed to include at least 234 participants.

### 2.3 Survey instruments

#### 2.3.1 General information questionnaire

We developed a questionnaire divided into two sections. The first section collected general demographic data, including gender, age, residence, marital status, living arrangements, educational level, occupation, per capita monthly household income, type of health insurance, social media engagement, participation in social activities, and community resource availability. The second section addressed disease-related information, encompassing the type of stroke, frequency of stroke incidents, dependency in activities of daily living (ADL), the number of combined diseases, and the duration of the illness. The self-designed questionnaire is based on multiple literatures regarding the influencing factors of loneliness, aiming to comprehensively assess the current status of loneliness among older adults with stroke and its related factors (Zuo et al., 2023; Meehan et al., 2023; Guo et al., 2023).

#### 2.3.2 Shortened Chinese Version of the Family Resilience Assessment Scale (FRAS-C)

We then utilized the Shortened Chinese Version of the Family Resilience Assessment Scale (FRAS-C), translated and introduced by Li et al. (2016), to assess family resilience among older adults with stroke. This scale comprises three dimensions: family belief system, family communication process, and family organizational model, featuring a total of 32 items. It employs a 4-point Likert scoring system, with overall scores ranging from 32 to 128, where higher scores indicate greater family resilience. The FRAS-C has been extensively applied in stroke research; previous studies reported a Cronbach's alpha coefficient of 0.97 (Yan et al., 2021), while this study found it to be 0.919. Additionally, the Construct Reliability (CR) was 0.846, and the Average Variance Extracted (AVE) was 0.6475.

#### 2.3.3 University of California, Los Angeles Loneliness Scale (UCLA)

In this study, we used the third edition of the University of California, Los Angeles Loneliness Scale (UCLA) (Russell, 1996), developed by Russell et al., to assess loneliness among older adults with stroke. This scale comprises 20 items and utilizes a 4-point Likert scoring system, with total scores ranging from 20 to 80 points. Specifically, scores below 28 signify a low level of loneliness, scores between 28 and 33 indicate a below-average level, scores from 34 to 38 represent an average level, scores from 39 to 44 reflect an above-average level, and scores exceeding 44 denote a high level of loneliness. Previous studies reported a Cronbach's alpha coefficient of 0.96 for this scale (Bottaro et al., 2023), while the current study achieved a significantly higher coefficient of 0.972, indicating excellent internal consistency.

#### 2.3.4 Social support rating scale (SSRS)

We utilized the Social Support Rating Scale (SSRS) developed by Xiao Shuiyuan et al. to evaluate the level of social support among older adults with stroke. The SSRS is widely recognized and used in China (Huang et al., 2020; Fang et al., 2022; Li et al., 2021). The scale comprises three dimensions: objective support, subjective support, and the utilization of social support, with a total of 10 items. Total scores range from 12 to 66, where higher scores indicate more favorable social support conditions. Specifically, scores below 35 signify low social support, scores between 35 and 45 indicate moderate support, and scores above 45 reflect high social support. Previous research indicated a Cronbach's alpha coefficient of 0.81 for this scale (Shen et al., 2022), while this study recorded a coefficient of 0.835. Additionally, the Construct Reliability (CR) was found to be 0.838, and the Average Variance Extracted (AVE) was 0.636.

#### 2.3.5 Family caregiving APGAR index (APGAR)

The APGAR index was used to assess older adults with stroke' subjective satisfaction with their family functioning. Developed by American scholar Smilkstein in 1978 (Smilkstein, 1978), the Family Caregiving APGAR Index Scale consists of five items: adaptation, partnership, growth, affection, and resolve. This scale utilizes a 3-point Likert scoring system, yielding total scores that range from 0 to 10, with higher scores reflecting more effective family functioning. Specifically, scores between 7 and 10 indicate good family function and high levels of care; scores ranging from 4 to 6 suggest moderate impairment and partial loss of family care; scores from 0 to 3 signify severe family function impairment and significant loss of care. Previous research reported a Cronbach's alpha coefficient of 0.88 for this scale (Liu T. L. et al., 2021), while the current study recorded a coefficient of 0.866. Additionally, the Construct Reliability (CR) was 0.867, and the Average Variance Extracted (AVE) was 0.567.

#### 2.3.6 Positive psychological capital questionnaire (PPQ)

To assess the psychological capital of older adults with stroke, we used the Positive Psychological Capital Questionnaire (PPQ) (Kuo et al., 2010; Wang and Xue, 2021). Developed by Zhang Kuo and colleagues, the PPQ was formulated after reviewing domestic and international literature and measurement tools. The questionnaire consists of 26 items across four dimensions: self-efficacy, resilience, optimism, and hope. Each item is rated on a 7-point Likert scale, with total scores ranging from 26 to 182, where higher scores reflect a more positive psychological state. Scores above 154 indicate a superior psychological state, 109 to 154 suggest a moderate level, and scores below 109 indicate a poor psychological state. Previous studies reported a Cronbach's alpha of 0.94 (Jeong and Kim, 2022), while this study achieved an alpha of 0.938. Additionally, the Construct Reliability (CR) was 0.917, and the Average Variance Extracted (AVE) was 0.733.

### 2.4 Survey methodology

The questionnaire survey method was used to gather data. Surveyors were trained to use uniform instructional language to introduce the requirements for completing the questionnaire, informing participants of the purpose of the study and the right not to participate or to withdraw from the survey at any time. Before conducting the survey, patients were informed of the study's purpose and significance, and their informed consent was obtained. Instructions for completing the questionnaire were explained, and patients filled it out according to their actual circumstances. For those unable to complete it independently, the Surveyors asked the questions and recorded the responses. To minimize bias, the Surveyors personally distributed and supervised the completion of all questionnaires, offering standardized explanations without leading questions or subjective input. Completed questionnaires were reviewed on-site, and any missing or illogical responses were addressed by the Surveyors before final collection. Before data entry, the questionnaires were rechecked for completeness, and those with more than 20% missing data were excluded. Two individuals were responsible for data entry and coding, and a random sample of entered data was cross-verified against the original responses to ensure accuracy.

### 2.5 Statistical methods

SPSS 25.0 and AMOS 24.0 were applied for data analysis after double checking. The data were expressed as frequencies, percentages, means and standard deviations. Using one-way analysis of variance to compare the differences in loneliness among older adults with stroke with different demographic and disease characteristics groups. Construct a multiple linear regression equation with statistically significant factors in one-way ANOVA as independent variables and loneliness score as dependent variable for analysis. Using Pearson correlation analysis to explore the correlation between family resilience, social support, family functioning, psychological capital and loneliness in older adults with stroke. The structural equation model was constructed using AMOS 24.0 software. The mediating effects were tested by the Bootstrap bias-corrected percentile method with 5,000 resamples. *P* < 0.05 indicated statistical significance.

## 3 Results

### 3.1 Demographic and disease characteristics of older adults with stroke

A total of 234 older adults with stroke were ultimately included in the study, with ages ranging from 60 to 86 years, and a mean age of 71.56 ± 6.88 years. In terms of demographic characteristics, there were 130 male patients, accounting for 55.6%, which was slightly higher than the number of females. Regarding marital status, the highest proportion was married patients, at 85.9% (201 cases). In terms of occupation, the largest proportion was farmers, at 72.2% (169 cases). Regarding educational level, 60.3% of patients had an elementary school education or lower. In terms of family economic status, 54.3% of patients had a per capita monthly family income of less than 2,000 yuan. Regarding the payment method for medical expenses, urban resident insurance was the primary method, accounting for 75.2% (176 cases). In terms of disease characteristics, 73.9% of patients had mild dependence in activities of daily living. The proportion of ischemic stroke patients was 96.2% (225 cases), and the majority of patients had their first stroke (175 cases, 74.8%). Detailed data are shown in Table 1.

**Table 1:** Demographic and disease characteristics.

**Item**	**Category**	***N***	**Rate (%)**	**Item**	**Category**	***N***	**Rate (%)**
Gender	Male	130	55.6	Type of health insurance	Urban Resident Insurance	176	75.2
	Female	104	44.4		Urban Employee Insurance	58	24.8
Age	60–70	103	44.0	Social media usage	None	0	0.0
	71–80	110	47.0		Yes	105	44.9
	>80	21	9.0		No	129	55.1
Place of residence	Urban	98	41.9	Community resource availability	Good	101	43.2
	Rural	136	58.1		Moderate	81	34.6
Marital status	Unmarried	0	0.0		Poor	52	22.2
	Married	201	85.9	Participation in social activities	Yes	90	38.5
	Divorced	1	0.4		No	144	61.5
	Widowed	32	13.7		Ischemic	225	96.2
Living arrangements	Living Alone	20	8.5	Stroke type	Hemorrhagic	9	3.8
	Living with Spouse	176	75.2	Frequency of stroke incidents	First episode	175	74.8
	Living with Children	22	9.4		Multiple episodes	59	25.2
	Living with Spouse and Children	13	5.6	Activities of daily living (ADL)	Mild dependence	173	73.9
	Other	3	1.3		Moderate dependency	44	18.8
Educational level	Elementary school	141	60.3		Severe dependency	17	7.3
	Junior High School	58	24.8	The number of combined diseases	0	13	5.6
	High School	32	13.7		1	48	20.5
	University or above	3	1.3		2	63	26.9
Occupation	Retired	59	25.2		≥3	110	47.0
	Farmer	169	72.2	The duration of the illness	Within 1 Month	65	27.8
	Self-employed	4	1.7		2–3 Months	58	24.8
	Other	2	0.9		4–5 Months	48	20.5
Per capita monthly household income	< 2,000 Yuan/Month	127	54.3		Over 6 Months	63	26.9
	2,000–5,000 Yuan/Month	65	27.8				
	> 5,000 Yuan/Month	42	17.9				

### 3.2 Total scores of family resilience, social support, family functioning, psychological capital, and Loneliness in older adults with stroke

The family resilience score of the older adults with stroke averaged (97.80 ± 16.88), while the social support score was (38.31 ± 8.74). The family functioning score averaged (7.06 ± 2.97), the psychological capital score was (119.85 ± 29.26), and the loneliness score averaged (35.07 ± 15.24).

### 3.3 Analysis of factors influencing loneliness in older adults with stroke

Univariate analysis was performed to examine the loneliness scores of older adults with stroke across various demographic and disease-related characteristics. The findings revealed statistically significant differences in loneliness based on factors such as place of residence, marital status, education level, per capita monthly household income, social media usage, Community resource availability, participation in social activities, stroke type, ADL, number of comorbidities, and illness duration (*P* < 0.05).

A multiple linear regression analysis was performed using the total loneliness score of older adults with stroke as the dependent variable and the statistically significant items from the univariate analysis as independent variables. The independent variables were coded as outlined in Table 2. The analysis revealed that key factors influencing loneliness among older adults with stroke included Per capita monthly household income, ADL, community resource availability, participation in social activities, and illness duration (*P* < 0.05). Detailed results are provided in Table 3.

**Table 2:** Assignment table for independent variables.

**Independent variable**	**Assignment method**
Duration of illness	< 1 month = 1, 2 month−3 month = 2, 4 month−5 month = 3, more than 6 months = 4
Place of residence	Urban = 1, rural = 2
Marital status	Unmarried = 1, Married = 2, Divorced = 3, Widowed = 4
Educational level	Elementary school = 1, Junior high school = 2, High school = 3, University or above = 4
Per capita monthly household income	< 2,000 yuan per month = 1, 2,000–5000 yuan per month = 2, > 5,000 yuan per month = 3
Social media usage	Yes = 1, No = 2
Community resource availability	Good = 1, Average = 2, Poor = 3
Participation in social activities	Yes = 1, No = 2
Type of stroke	Ischemic stroke = 1, Hemorrhagic stroke = 2
ADL	Mild dependency = 1, Moderate dependency = 2, Severe dependency = 3
The number of combined diseases	0 case = 1, 1 case = 2, 2 case = 3, more than 3 case = 4

**Table 3:** Results of multiple linear regression analysis of loneliness (*N* = 234).

**Item**	**Regression coefficient**	***t/F* value**	***P***	**VIF**
Place of residence	−0.918	−0.594	0.553	1.308
Marital status	0.396	0.385	0.7	1.120
Educational level	1.245	1.184	0.238	1.477
Per capita monthly household income	−2.58	−2.236	0.026	1.767
ADL	5.031	3.843	< 0.001	1.615
Social media usage	1.769	1.044	0.298	1.793
Community resource availability	6.691	5.862	< 0.001	1.638
Participation in social activities	4.918	2.793	0.006	1.082
Type of stroke	2.283	0.632	0.528	1.408
The number of combined diseases	0.294	0.391	0.696	1.146
Duration of illness	−3.45	−5.858	< 0.001	1.195

*F* = 33.779, *P* < 0.001, *R*^2^ = 0.626, adjusted *R*^2^ = 0.607; VIF, variance inflation factor.

### 3.4 Correlation between family resilience, social support, family functioning, psychological capital, and loneliness in older adults with stroke

Pearson correlation analysis showed a significant negative correlation between loneliness and family resilience, social support, family functioning, and psychological capital in older adults with stroke (*P* < 0.01). Furthermore, family resilience, social support, family functioning, and psychological capital were positively correlated with one another (*P* < 0.01). Detailed correlation coefficients are presented in Table 4.

**Table 4:** Correlation analysis of family resilience, social support, family functioning, psychological capital, and loneliness (*r*).

**Project**	**Loneliness**	**Family resilience**	**Social support**	**Family functioning**	**Psychological capital**
Loneliness	1				
Family resilience	−0.738^**^	1			
Social support	−0.715^**^	0.618^**^	1		
Family functioning	−0.745^**^	0.604^**^	0.614^**^	1	
Psychological capital	−0.684^**^	0.520^**^	0.542^**^	0.533^**^	1

^**^P < 0.01.

### 3.5 Construction of a structural equation model for loneliness

**Figure 2:**
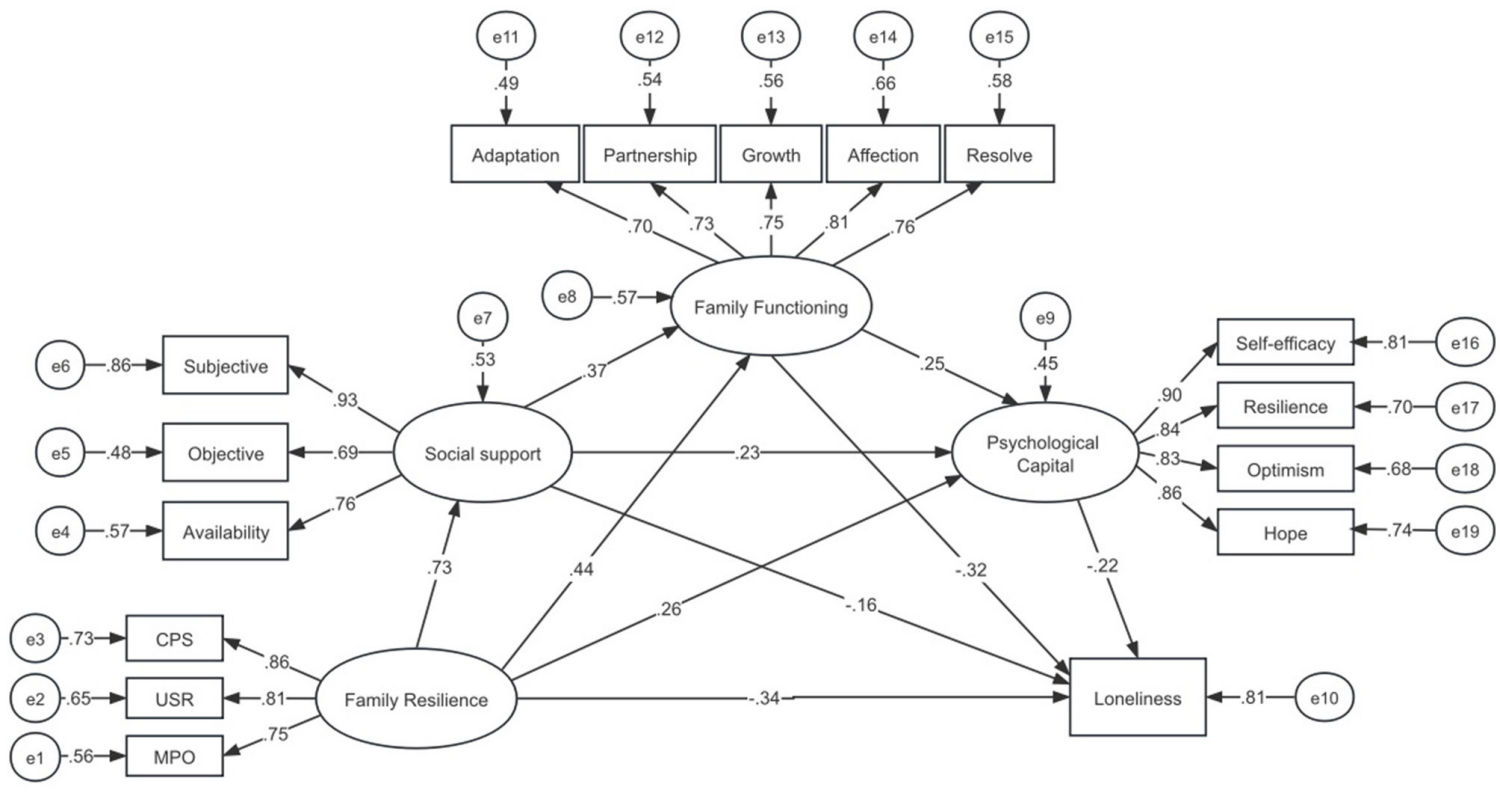
Chain mediation model diagram of social support, family function, and psychological capital in family resilience and loneliness.

**Figure 3:**
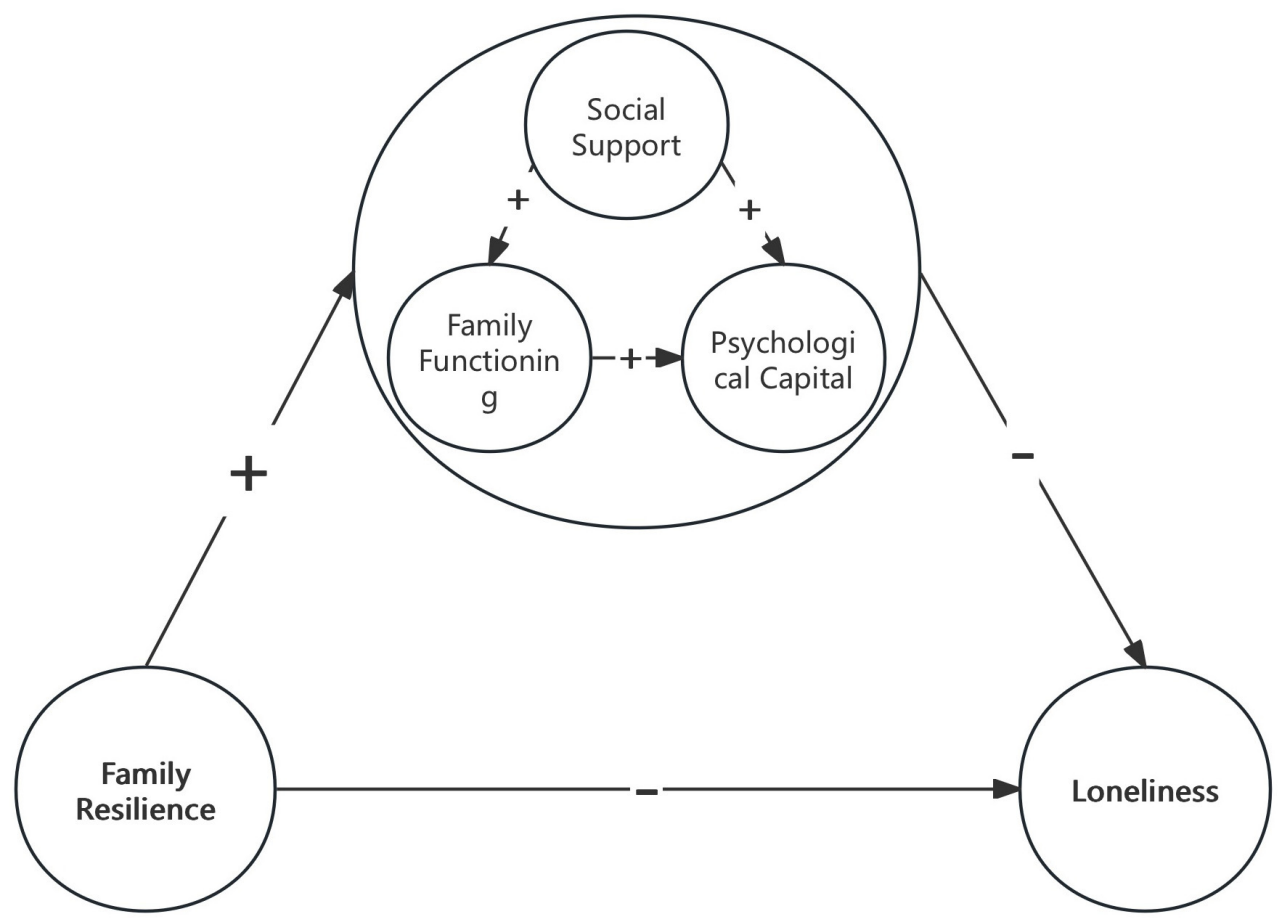
Conceptual diagram.

With family resilience as the independent variable and loneliness as the dependent variable, a structural equation model was constructed with social support, family functioning, and psychological capital as mediating variables. Data analysis was conducted using Amos 24.0 Its main fit indices are: χ^2^*/df* = 1.148 < 3; RMSEA = 0.025 < 0.05; GFI = 0.946 > 0.9; AGFI = 0.923 > 0.9; CFI = 0.994 > 0.9, indicating a good model fit (Figure 2). We constructed a conceptual diagram to summarize the Structural Equation Model (SEM) and the mediating pathways (Figure 3). The Bootstrap method was used to repeatedly sample 5,000 times to calculate the 95% confidence interval for mediating effect tests. The results showed that family resilience has a direct negative predictive effect on loneliness (β = −0.342, *P* < 0.001); social support has a negative predictive effect on loneliness (β = −0.157, *P* < 0.001), and it plays a partial mediating role between family resilience and loneliness, with a mediating effect value of −0.114, accounting for 14.65% of the total effect; family functioning has a negative predictive effect on loneliness (β = −0.319, *P* < 0.001), and it plays a partial mediating role between family resilience and loneliness, with a mediating effect value of −0.140, accounting for 17.99% of the total effect; psychological capital has a negative predictive effect on loneliness (β = −0.216, *P* < 0.001), and it plays a partial mediating role between family resilience and loneliness, with a mediating effect value of −0.057, accounting for 7.33% of the total effect; social support and family functioning have a chain mediating effect between family resilience and loneliness, with a mediating effect value of −0.086, accounting for 11.05% of the total effect; family functioning and psychological capital have a chain mediating effect between family resilience and loneliness, with a mediating effect value of −0.024, accounting for 3.08% of the total effect; social support, family functioning, and psychological capital have a chain mediating effect between family resilience and loneliness, with a mediating effect value of −0.015, accounting for 1.93% of the total effect. The confidence intervals corresponding to the model tests all did not include 0, indicating the existence of mediating effects and the establishment of chain mediation (Tables 5, 6).

**Table 5:** Regression analysis of family resilience, social support, family functioning, psychological capital, and loneliness.

**Dependent variable**	**Predictor variable**	***r* ^2^**	**β**	**SEs**	***t***	***P***	**LLCI**	**ULCI**
Social support	Family resilience	0.535	0.731	0.057	8.783	*P* < 0.001	0.64	0.806
Family functioning	Family resilience	0.569	0.440	0.016	4.289	*P* < 0.001	0.235	0.626
	Social support		0.371	0.023	3.703	*P* < 0.001	0.163	0.554
Psychological capital	Family resilience	0.453	0.264	0.289	2.395	0.017	0.017	0.471
	Social support		0.234	0.392	2.285	0.022	0.032	0.466
	Family functioning		0.250	1.689	2.485	0.013	0.053	0.476
Loneliness	Family resilience	0.806	−0.342	0.368	−4.934	*P* < 0.001	−0.506	−0.188
	Social support		−0.157	0.485	−2.501	0.012	−0.302	−0.031
	Family functioning		−0.319	2.183	−4.962	*P* < 0.001	−0.467	−0.182
	Psychological capital		−0.216	0.098	−4.485	*P* < 0.001	−0.312	−0.125

**Table 6:** Path and effect analysis among variables in the model (*N* = 234).

**Project**	**Effect size**	**Standard error**	**Bootstrap 95%** ***CI***		**Proportion of total effect (%)**
Total effect	−0.778	0.029	−0.866	−0.751	100
Direct effect	−0.342	0.081	−0.506	−0.188	43.96
Total indirect effect	−0.436	0.065	−0.606	−0.345	56.04
X-M1-Y	−0.114	0.049	−0.222	−0.024	14.65
X-M2-Y	−0.140	0.049	−0.263	−0.062	17.99
X-M3-Y	−0.057	0.027	−0.117	−0.01	7.33
X-M1-M2-Y	−0.086	0.031	−0.161	−0.036	11.05
X-M2-M3-Y	−0.024	0.013	−0.061	−0.006	3.08
X-M1-M2-M3-Y	−0.015	0.008	−0.036	−0.004	1.93

X, family resilience; M1, social support; M2, family functioning; M3, psychological capital; Y, loneliness.

## 4 Discussion

### 4.1 Analysis of the current situation of older adults with stroke

The findings of this study reveal that the overall loneliness score for older adults with stroke was (35.07 ± 15.24), indicating a moderate level of loneliness. Notably, 117 patients (50%) reported experiencing moderate to severe loneliness, a figure that surpasses the incidence of moderate to severe loneliness among the general elderly population as reported by Ying et al. (2021). This suggests that loneliness among older adults with stroke may be more pronounced than in the broader elderly demographic. Studies have shown that loneliness has adverse effects on the health of the elderly. It has been identified as a risk factor for suicide, cardiovascular and cerebrovascular diseases, all-cause morbidity and mortality in the elderly, and can also lead to mental health issues such as dementia and depression (McClelland et al., 2020; Prince et al., 2015; Hakulinen et al., 2018; Isik et al., 2021). Therefore, healthcare professionals should pay attention to the loneliness of older adults with stroke.

This study found that all older adults with stroke experienced varying degrees of loneliness, but the severity was lower than that reported in Hollands L's cross-sectional survey (Hollands et al., 2024). The reason for this difference may be that his study included a large number of patients with aphasia, whose impaired language function may lead to a loss of connection with a broader social network, thereby increasing their sense of loneliness (Hilari et al., 2010). Additionally, his survey was conducted during the COVID-19 pandemic, and the visiting restrictions during the pandemic, as an external factor, further weakened the social connections of the patients, exacerbating their loneliness (Su et al., 2023). In contrast, in this study, 73.9% of the patients undergoing home-based rehabilitation had mild dependency in their self-care abilities. They may have been more independent in their daily lives. Although they also had certain physical functional impairments, they were recovering in a familiar environment with more interaction with their family members and a relatively intact social support network, which may have alleviated their loneliness to some extent (Bergersen et al., 2024). Therefore, the loneliness of older adults with stroke is closely related to their family environment. While healthcare professionals are committed to the physical rehabilitation of patients, they should also pay close attention to their mental health status. By building communication bridges to enhance the interaction between patients and their families, and encouraging family members to provide more emotional and practical support, the loneliness of patients can be reduced, their mental health status can be improved.

### 4.2 Analysis of the current status and influencing factors of loneliness in older adults with stroke

This study identified several factors influencing loneliness in older adults with stroke through multivariate analysis, including ADL, Per capita monthly household income, participation in social activities, community resource availability, and duration of illness. The findings suggest that older stroke patients with limited daily activity capacity tend to experience higher levels of loneliness, aligning with research by Pan et al. (2022). This may be attributed to a decline in daily activity capacity, which restricts patients' ability to engage in social activities such as shopping and dining, thereby diminishing their interactions with the outside world and increasing the risk of social loneliness. Most stroke patients are accompanied by a certain degree of disability after onset, which leads to loss of self-identity and physical dysfunction, resulting in a sense of shame (Huang et al., 2024; Kariasa et al., 2024). Due to the decline in daily activity ability, stroke patients often require the care and assistance of multiple family members, leading to feelings of stigma and self blame (Hughes and Cummings, 2020; Zhang Y. et al., 2023). The shame and guilt of stroke patients exacerbate their sense of loneliness by weakening their social motivation, reducing their sense of self-worth, and decreasing social interaction.

Family economic status plays a crucial role in providing material security post-illness and significantly affects patients' treatment decisions and rehabilitation attitudes (Rajsic et al., 2019). Stroke patients with lower per capita family income often worry about treatment costs, and this financial strain can lead to a passive approach toward recovery and social engagement. Additionally, economic stress may precipitate psychological issues such as anxiety, depression, and low self-esteem, which are closely linked to loneliness (Peltzer and Pengpid, 2019). Social participation is vital for the elderly to maintain a positive outlook on life and a sense of self-worth. However, limited social activities and inadequate community resources can hinder social interaction among older adults with stroke, diminishing their sense of participation and leading to a regression in social roles and self-worth, ultimately contributing to loneliness (Elayoubi et al., 2023).

Moreover, this study observed a significant negative correlation between the duration of illness and loneliness in older adults with stroke, which slightly contrasts with Guo et al. (2024) longitudinal findings on first-time stroke patients. This discrepancy may stem from better functional outcomes in patients whose illness has lasted over 6 months in this survey. Following the traumatic event of a stroke, patients often experience intense loneliness initially due to the shock and unfamiliarity of their circumstances. However, as recovery progresses and patients adapt to their condition and regain physical function, they may develop greater confidence, facilitating a gradual return to normal social interactions. This improvement in social participation may, in turn, help alleviate feelings of loneliness.

### 4.3 The impact of family resilience on loneliness in older adults with stroke

Family resilience influences depressive moods through two primary mechanisms: enhancing individual resilience and bolstering social support, highlighting its significant role in promoting and safeguarding mental health (Walsh, 2003). The family resilience theory posits that families function as dynamic systems capable of integrating both internal and external resources to adapt to stressors and adversity. Externally, a well-structured family environment is essential for alleviating loneliness among older adults with stroke. It offers consistent material support, emotional comfort, and opportunities for social interaction, which collectively enhance the patient's sense of belonging and self-worth, thereby effectively mitigating the detrimental effects of loneliness. Internally, the family's belief system acts as a motivational force, encouraging patients to adopt a positive outlook, which can alleviate stress arising from negative emotions and help maintain psychological wellbeing. Furthermore, a robust family communication framework enhances the level of care provided to the patient and fosters the mobilization of positive psychological resources within the individual. Together, these elements create a strong psychological support network that helps combat feelings of loneliness (Li K. et al., 2024; Lima et al., 2023; Lou and Ng, 2012).

The findings of this study reveal that older adults with stroke scored an average of 97.80 ± 16.88 on the FRAS-C, indicating a moderate level of family resilience. A closer examination of the dimension scores shows that both family belief system and family organizational model received lower average ratings. This suggests that older adults with stroke and their families may struggle with sustaining a positive attitude and seeking external social support effectively. Medical professionals should focus on enhancing interactions between patients and their families, employing various intervention strategies to bolster family resilience. First, communication skills training for medical staff can improve the effectiveness of dialogue between patients and their families, facilitating better information exchange. Second, guiding families toward a problem-solving approach, such as through family meetings, can encourage collective discussions to address challenges, thereby fostering the patient's intrinsic motivation and promoting positive outlook. Additionally, healthcare providers should actively work to expand the social resources available to families of older adults with stroke, educating them on how to access these resources. This approach can enhance families' awareness and utilization of social support, ultimately aiding in the psychosocial recovery of the patients.

### 4.4 The mediating role of social support between family resilience and loneliness

Family resilience is a strong positive predictor of social support levels (β = 0.73, *P* < 0.001). The mediating effect of social support in the relationship between family resilience and loneliness is measured at 0.1147, which corroborates the findings of Zhao et al. (2018). Families exhibiting high resilience, particularly when confronting challenges posed by illnesses such as stroke, tend to demonstrate keen awareness and effective engagement with their social support networks. They systematically mobilize resources from relatives and friends, thoroughly explore community services, and leverage medical resources to establish a robust social network for the patient. This proactive approach not only enhances the patient's social integration but also provides a solid foundation for effective disease management.

Social support is a significant negative predictor of loneliness (β = −0.16, *P* < 0.05), aligning with findings from several studies (Farhang et al., 2024; Yu et al., 2024; Wen et al., 2024). Research indicates that patients who experience a sudden stroke are often susceptible to acute stress disorder (Unal et al., 2017). Access to community support resources can assist these patients in establishing a stable social network, thereby alleviating feelings of loneliness and self-isolation. The emotional comfort and practical aid offered by relatives and friends in daily life can mitigate the stress associated with the illness, enabling patients to maintain a positive emotional outlook throughout their recovery. This support enhances their self-identity and self-efficacy, ultimately reducing the risk of experiencing loneliness.

### 4.5 The mediating role of family function between family resilience and loneliness

Family resilience positively influences the family functioning of older adults with stroke (β = 0.44, *P* < 0.001), with a mediating effect value of 0.1408 observed between family resilience and loneliness. Enhancements in family resilience are typically accompanied by improved communication and support among family members, fostering a positive family environment. This supportive atmosphere allows family members to collaborate in overcoming difficulties, effectively addressing life's challenges. Consequently, family cohesion and functionality are strengthened (Chen et al., 2018). Additionally, family functioning serves as a negative predictor of loneliness (β = −0.32, *P* < 0.001), a correlation corroborated by multiple studies (Qiu et al., 2024; Qian et al., 2022). Within a nurturing family setting, older adults with stroke often feel a heightened sense of psychological security and social belonging—critical psychological resources that combat loneliness. Conversely, when family care is inadequate, patients may perceive themselves as neglected and isolated, struggling to attain emotional fulfillment and support. This emotional shortfall can exacerbate their feelings of psychological alienation.

### 4.6 The mediating role of psychological capital between family resilience and loneliness

Family resilience is a positive predictor of the psychological capital of older adults with stroke (β = 0.26, *P* < 0.05), with a mediating effect value of 0.0572 identified between family resilience and loneliness. Families characterized by strong resilience not only enhance the patient's self-efficacy, instilling greater confidence in their ability to tackle rehabilitation challenges, but also cultivate an environment imbued with hope and optimism. Such an atmosphere is essential for alleviating anxiety and depression, thereby increasing the likelihood that patients will adopt a resilient mindset, adapt swiftly to adversity, and seek solutions to their problems, which in turn reinforces their psychological capital (Li et al., 2019, 2018). The enhancement of psychological capital not only promotes the patient's mental wellbeing but also translates into positive social behaviors. This study indicates that psychological capital negatively predicts loneliness in older adults with stroke (β = −0.22, *P* < 0.001). Prior research has shown that individuals with high psychological capital are more likely to engage in social activities and cultivate healthy social relationships, significantly reducing feelings of loneliness (Ren and Ji, 2019). Consequently, family resilience can indirectly diminish loneliness in older adults with stroke by fostering their psychological capital, which is crucial for their long-term rehabilitation.

### 4.7 The chain mediating role of social support, family function, and psychological capital between family resilience and loneliness

This study explores the intricate relationship between family resilience and the loneliness experienced by older adults with stroke, revealing a distinct pathway of influence: family resilience → social support → family functioning → psychological capital → loneliness. It demonstrates that family resilience can indirectly impact the loneliness of older adults with stroke through this interconnected mechanism. Specifically, older stroke patients with strong family resilience have a more solid social support network, enabling them to receive more assistance from friends, relatives, the community, and professional institutions. The intervention of this support helps to alleviate family conflicts and tension caused by the illness. Family members reposition their roles, coordinate internal relationships, and jointly confront challenges, thereby maintaining family functions (Acoba, 2024). The positive interactions among family members play a crucial role in enhancing the patient's psychological capital. Studies have shown that encouragement and support from family members can significantly increase the patient's level of hope (Yang et al., 2022), which is of great importance for reducing loneliness.

In conclusion, this study elucidates the specific pathways and mechanisms through which family resilience influences loneliness. Compared to prior research, it offers deeper insights into the interactive relationships among various components within the family system and their synergistic effects on the mental health of patients. Additionally, it presents a fresh perspective for family interventions, indicating that bolstering family resilience and enhancing family functioning can facilitate the development of patients' social support networks and the growth of psychological capital, effectively preventing and alleviating loneliness in older adults with stroke.

### 4.8 Importance of family reliance

Based on the theory of family resilience, the family, as a dynamic system, has the capacity to integrate internal and external resources to adapt to stress and adversity (Walsh, 2016, 2003). In terms of external resources, a well-organized family structure can expand the patient's social support network and enhance their sense of belonging and self-worth by providing material support, emotional comfort, and opportunities for social interaction (An et al., 2024; Nshimyumuremyi et al., 2023). In terms of internal resources, the family belief system, as an internal force guiding the patient to live positively, can alleviate negative emotions and maintain a positive psychological state (Zhu et al., 2024). A good family communication system can further enhance family care by promoting understanding and trust among family members through open and sincere communication, creating a warmer and more supportive family environment, which helps to mitigate the negative impact of loneliness (Favotto et al., 2019; Chiao et al., 2021).

Therefore, to address the issue of loneliness in older adults with stroke, a family systems perspective should be adopted. On the one hand, intrinsic resilience can be enhanced through methods such as family belief reconstruction and communication skills training. On the other hand, external support acquisition can be optimized through resource linking and caregiving guidance. Clinical healthcare providers should offer family-participatory nursing interventions to help patients better cope with the challenges of illness, thereby reducing their sense of loneliness.

### 4.9 Innovation in research

Currently, there is limited research on the sense of loneliness among older adults with stroke, and the analysis of its influencing factors is not comprehensive. Meanwhile, the role of family in improving loneliness is often overlooked. This study innovatively incorporates the well-researched influencing factors of loneliness, delving into the factors that contribute to loneliness among stroke patients. Based on the social ecosystem theory and family resilience theory, this study constructs a theoretical framework, recognizing loneliness as a result of the combined effects of individual, family, and social environmental factors. It analyzes the direct relationships and mediating effects between family resilience, psychological capital, family functioning, and social support on loneliness among older adults with stroke, highlighting the importance of family in preventing and improving loneliness among older adults with stroke.

## 5 Conclusion

This study highlights that loneliness is an issue that cannot be ignored in the rehabilitation process of older adults with stroke and requires high attention from healthcare professionals and society. The power of the family plays an important role in alleviating loneliness. When healthcare professionals develop rehabilitation and care plans for older adults with stroke, they should place greater emphasis on the role of the family. By enhancing family resilience, they can improve communication efficiency among family members, strengthen their ability to collaborate in problem-solving, and expand access to social resources. These measures are crucial for alleviating the patients' loneliness, and promoting their mental health.

This study has several limitations. First, the reliance on convenience sampling from a single geographic area may compromise generalizability due to potential regional biases, such as socioeconomic or cultural homogeneity. Second, although structural equation modeling was applied, the cross-sectional design precludes establishing causality between family resilience and loneliness. Third, unmeasured confounding factors, such as cognitive function, sleep quality, and healthcare accessibility, may influence the observed associations. Fourth, the general questionnaire did not directly include content related to family resilience. This has led to a lack of close and intuitive connection between family resilience and other variables in the overall structure of the questionnaire.

Future studies should prioritize multicenter probability sampling to enhance representativeness and longitudinal designs to establish causality. Additionally, incorporating neuropsychological and healthcare variables into the analytical models will elucidate potential mechanisms. Finally, intervention studies targeting mediating pathways, such as family resilience programs, are needed to translate research findings into strategies for alleviating loneliness among older adults with stroke. Addressing these gaps will advance the theoretical framework and evidence-based care for this vulnerable population.

## Data Availability

The raw data supporting the conclusions of this article will be made available by the authors, without undue reservation.
